# Morbidity and mortality in a population of patients affected by heart failure and chronic obstructive pulmonary disease: an observational study

**DOI:** 10.1186/s12872-018-0986-y

**Published:** 2019-01-16

**Authors:** Eugenio Roberto Cosentino, Matteo Landolfo, Crescenzio Bentivenga, Luca Spinardi, Daniela Degli Esposti, Arrigo Francesco Cicero, Rinaldo Miceli, Virna Bui, Emanuela Berardi, Claudio Borghi

**Affiliations:** 1 Cardio–Thoracic–Vascular Department, Policlinico S. Orsola-Malpighi, Università di Bologna, Via Albertoni 15, 40138 Bologna, Italy; 2 Cardiology Department, Hospital S. Valentino, Treviso, Montebelluna Italy

**Keywords:** Chronic obstructive pulmonary disease, Elderly, Heart failure, Indacaterol/glycopirronium

## Abstract

**Background:**

Chronic obstructive pulmonary disease (COPD) and heart failure (HF) often coexist. Moreover, elderly patients suffering from HF have a higher incidence of COPD, which further complicates their clinical condition. Indacaterol/glycopirronium has shown benefits in the treatment of COPD, with few cardiologic adverse effects. We evaluated the safety and efficacy of this therapy in patients with history of HF.

**Methods:**

We enrolled 56 patients with a history of HF (New York Heart Association [NYHA] classes II and III) and stable COPD. We evaluated blood samples, clinical assessment, echocardiograms and basal spirometry at baseline and after 6 months of therapy with indacaterol/glycopirronium. In addition, the number of re-hospitalizations during the treatment period was evaluated.

**Results:**

The treatment was well tolerated. Brain natriuretic peptide (BNP) levels were significantly reduced compared with baseline (*p* < 0.001) after 6 months of treatment, and a higher percentage of patients improved their clinical status compared with baseline (*p* < 0.001). Minor changes were noted in the hemodynamic and metabolic parameters. Significant improvements in the echocardiographic parameters were noted in HF with reduced ejection fraction (HFrEF) patients. All respiratory parameters (forced expiratory volume in 1 s [FEV1], FEV1/forced vital capacity [FVC] ratio and COPD Assessment Test [CAT] scores) improved significantly (*p* < 0.001). No hospitalizations owing to HF or COPD exacerbation occurred. One patient died of respiratory failure.

**Conclusion:**

Indacaterol/glycopirronium was well-tolerated and effective in the treatment of COPD in this cohort of patients with a history of HF. Further studies are needed to clarify whether this compound can have a direct role in improving overall cardiovascular function.

## Background

Chronic obstructive pulmonary disease (COPD) is a preventable and treatable disease characterised by persistent respiratory symptoms and airflow limitation. It is a major cause of morbidity and mortality throughout the world [1–4]. COPD and heart failure (HF) both share a high rate of prevalence, morbidity and mortality among the population [1, 2]. Quite often, COPD and HF may coexist in the same patient, with HF being one of the most frequent comorbidities in patients with COPD, with consequent major issues regarding proper diagnosis and treatment of both conditions.

The relationship between COPD and cardiovascular events is still unclear: systemic inflammation typical of COPD is thought to play a role in accelerating atherosclerosis and causing coronary artery disease and subsequently HF. HF is diagnosed if clinical signs and symptoms demonstrated in patients are confirmed by instrumental examinations; the presence of pulmonary disease could affect the presentation of clinical symptoms and signs according to the Framingham criteria. For instance, exertional dyspnoea, cough and paroxysmal dyspnoea are typical of both HF and COPD.

Natriuretic peptides (NPs) are known prognostic biomarkers in patients with HF. In particular, Pavasini et al. recently showed that NT-proBNP values are related to increased risk of all-cause mortality in COPD patients both with and without exacerbation and it is now considered a reliable predictive biomarker of poor prognosis in patients with COPD [3].

The effective prevalence of COPD is difficult to obtain: estimates vary according to the subpopulation involved, criteria applied for diagnosis, examinations and measurements performed, and surveillance programs [2]. For instance, geographical differences correlate with age and risk factors exposure, such as cigarettes smoking [2, 4].

At present, no clinical trial has systematically examined the respiratory performances of patients with HF. However, in the Cardiovascular Health Study, the prevalence of COPD in patients with HF was higher than in the general population (20 vs 13%; *p* = 0.001) [5], and these data were corroborated by the recent results of the European Society of Cardiology Heart Failure Long-Term Registry [6]. Moreover, in North America, this prevalence varies from 11 to 52%, whereas in Europe it ranges between 9 and 41% [7]. Of note, COPD prevalence in patients with HF increased until the age of 75 years, and then it decreased due to reduced survival in the elderly [8–11]. Importantly, the prevalence of COPD is higher in HF patients who show a preserved ejection fraction (HFpEF) [13] being approximately 20% in elderly patients without echocardiographic signs of right ventricular dysfunction or chronic cor pulmonale [12, 13].

Cigarette smoking, one of the most important risk factors of COPD, increases the likelihood of developing HF by 50%, which is 4.5-times higher in patients with COPD compared with the control (patients without COPD) [14]. For this reason, patients with suspected COPD should be evaluated carefully until confirmation or exclusion of ventricular dysfunction. For instance, in a recent systematic review, reduction of left ventricular ejection fraction (LVEF) in patients with COPD was noted in 10–46% of patients depending on estimates [15]. Mortality rates are also higher in patients with concomitant HF and COPD [16].

All these data should alert clinicians to carefully evaluate patients presenting with shortness of breath, in order to assess their ventricular function and properly start the best treatment depending on the results of both pulmonologist and cardiologist consultations.

In 2017, the GOLD report recommended a monotherapy with a long-acting beta-antagonist (LABA) or long-acting muscarinic-antagonist (LAMA) for symptomatic and not exacerbating patients with COPD [2]. If the patient continues to experience dyspnoea, or if dyspnoea is severe, GOLD recommends the use of LABAs and LAMAs in combination regardless of his exacerbation risk. However, there is a major need for improving treatment of COPD in patients with concomitant HF [17]. Newer drugs such as the indacaterol-glycopirronium (IND/GLY) combination have shown major results in COPD patients, with a good safety and tolerability profile in cases of coexistence of COPD and HF [18]. IND/GLY is a fixed-dose combination (FDC) of indacaterol, an inhaled long-acting β2-agonist (LABA), and glycopyrronium, an inhaled long-acting muscarinic antagonist (LAMA), developed as a maintenance bronchodilator treatment for patients with COPD. It provides dual bronchodilation which may be beneficial above and beyond both the monotherapies and LABA/ICS combinations, for improving lung function in patients with COPD, in particular for non-exacerbating patients. Briefly, glycopyrronium is a highly potent, competitive muscarinic receptor antagonist that binds to muscarinic receptors in bronchial smooth muscle and inhibits acetylcholine-mediated bronchoconstriction, binding with high affinity to M_1–3_ receptors and 4–5-fold higher selectivity for M_1_ and M_3_ subtypes over M_2._ It also shows faster dissociation from M_2_ than from M_1_ and M_3_ when compared with tiotropium [19]. On the other hand, indacaterol is an ultra-long-acting β-2 agonist. Its duration of action is approximately 24 h, allowing for once-daily administration. It stimulates adrenergic beta-2 receptors in the smooth muscle of the airways, causing relaxation of the muscle, which thereby increases the diameter of the airways. Its long action is due to the high affinity to the lipid raft domains in the airway membrane; that cause a slow dissociation from the receptors. Its onset of action is very fast, occurring within 5 min. The pharmacological effects of beta2-adrenoceptor agonist drugs, including indacaterol, are at least in part attributable to the stimulation of intracellular adenyl cyclase, the enzyme that catalyzes the conversion of adenosine triphosphate (ATP) to cyclic-3′, 5′-adenosine monophosphate (cyclic monophosphate). Increased cyclic AMP levels cause relaxation of bronchial smooth muscle [20].

Given the GOLD recommendations, indacaterol/glycopirronium is being increasingly used in clinical practice, also in patients with concomitant COPD and HF. Thus, the aim of our analysis was to investigate its efficacy and tolerability/safety in a population of outpatients with stable COPD and coexisting HF.

## Methods

**Fig. 1:**
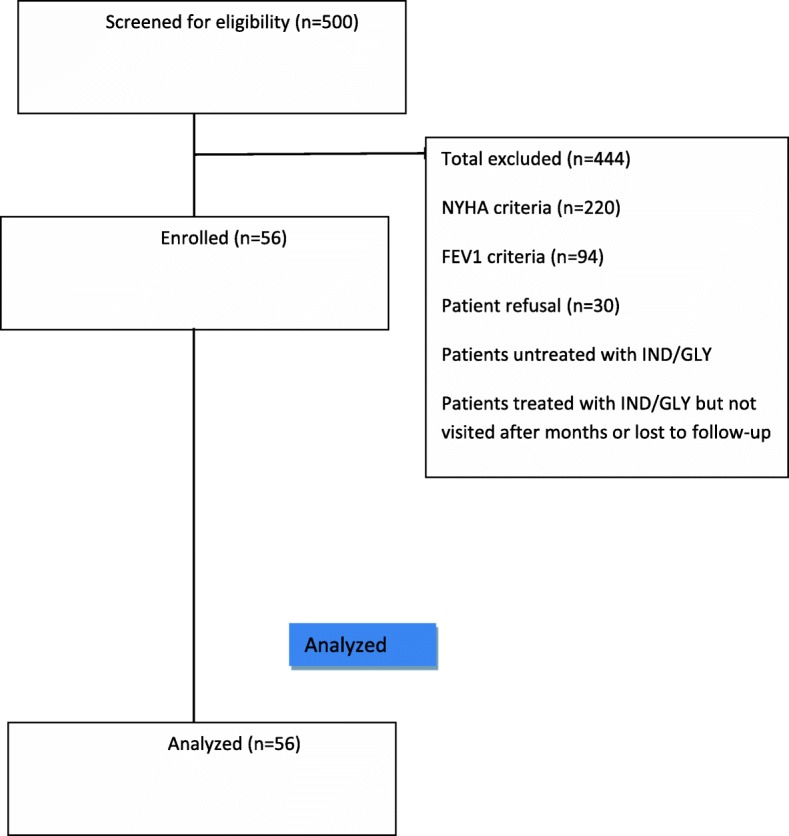
Study design and patients’ enrolment

Among all patients who were afferent to our clinic (*n* = 500), we identified a cohort of patients with concomitant COPD and stable HF in New York Heart Association (NYHA) class II–III, eligible to receive IND/GLY. Among them, 220 were excluded from final analysis due to NYHA criteria, 94 patients were excluded due to FEV1 criteria, and 30 patients refused consent to participate in the study (Fig. 1). Thus, the final analysis retrieved data for 56 subjects (from the database of our outpatient service dedicated to the diagnosis and treatment of heart failure, in the Cardio–Thoraco–Vascular Department of the University of Bologna) with stable COPD and HF that have been treated with IND/GLY 110/50 μg once daily for at least 6 months. Data were analysed retrospectively, with patients followed-up as per clinical practice.

Primary endpoints of the study were changes in the haematic, echocardiographic, respiratory parameters as well as clinical improvement. Blood samples were taken after a period of 12-h fasting, and the dosing of all immunochemistry was standardized by the central laboratory of S. Orsola-Malpighi University Hospital. Systemic blood pressure was measured using standard sphygmomanometer with appropriate cuff dimensions, in patients resting at least 10 min before measurements. Three consecutive blood pressure measurements at 5-min intervals were obtained, and then averaged to obtain a mean value. Echocardiographic and Doppler measurements of the left ventricle were made according to established procedures [21], in the left lateral decubitus, at end expiration. A basal spirometry and a COPD Assessment Test (CAT) were also performed. This is a new scoring system for COPD patients, which provides a simple method for assessing the impact of COPD on the patient’s health. Validation studies showed that it is similar to St. George’s Respiratory Questionnaire (SGRQ) in terms of efficacy [22, 23]. The CAT is a standard and validated test containing eight items for the evaluation of the impact of COPD on health status [23]. However, FEV_1_ remains essential to establish a diagnosis and to confirm the severity of airway obstruction in symptomatic COPD patients [24]. CAT has a scoring range of zero to 40. The CAT score classifies patients into four groups of low, medium, high and very high risk based on the impact of their disease over their health statusAll measurements were obtained at baseline and after 6 months of treatment with the IND/GLY combination.

Informed, written consent was obtained from all participants. The study was evaluated by the ethical board of the S. Orsola-Malpighi University Hospital.

### Statistical analysis

Statistical analysis was performed with SPSS 19.0 software, considering 2-side 0.05 level of significance. A full descriptive analysis of categorical and continuous variables was carried out. Pre- and post-treatment patient characteristics were compared with chi-square followed by the Fisher exact test (categorical data), student t-test for paired samples (continuous normally distributed data) or Mann-Whitney-U test (continuous not normally distributed variables). A univariate, as well as a multivariate, analysis was performed. Among the variables included, we report age, systemic blood pressure, heart rate, serum potassium and sodium concentrations, serum creatinine, BNP, NYHA class, LVEF and other echocardiographic signs of systolic dysfunction.

## Results

As shown in Fig. 1, the analysis involved 56 patients (61% males, mean age: 76 ± 9 years, age range: 49–91 years). At enrolment, 41% of the patients were in NYHA class II, and 59% in NYHA class III. 16 patients had an ejection fraction (EF) ≤40% (HFrEF), 40 patients had an EF ≥50% (HFpEF). HF was ischemic in aetiology in 27% of the population, mixed hypertensive/ischaemic in 7% of patients, valvular in 7% of cases, and hypertensive in the remaining 59%. The main comorbidities in our population were type 2 diabetes mellitus in 29% of patients, dyslipidaemia in 61%, acute stroke in 9%, chronic kidney disease in 57% and atrial fibrillation in 48% of patients. In the previous 12 months for the sample of evaluated patients, the number of hospitalizations for HF was 84, with a total of 485 days of hospital stay.

### Clinical evaluation

Mean systolic blood pressure was 128 ± 15 mmHg, with a mean diastolic blood pressure of 75 ± 10 mmHg. The mean resting heart rate was 75 ± 14 bpm. Mean values of all hemodynamic and metabolic (hematic) parameters considered are reported in Table 1, where they are compared to the same values obtained after 6 months of treatment. Active cardiologic and respiratory therapies at baseline and during follow up are listed in Table 2. Table 3 shows the mean values of the most relevant echocardiographic parameters in both subgroups of patients with HFrEF and HFpEF, and consequent changes over time, whereas forced expiratory volume at 1 s (FEV1) and Tiffenau Index are listed in Table 4, at baseline and after 6 months. At baseline, mean CAT score was 19 ± 4 points.

**Table 1:** Hemodynamic and metabolic parameters at baseline and after 6 months of treatment

Hemodynamic and metabolic parameters	Baseline	6 months
SBP (mmHg)	128 ± 15	131 ± 18
DBP (mmHg)	75 ± 10	74 ± 9
HR (bpm)	70 ± 14	70 ± 16
LVEF (%)	53 ± 13	54 ± 13
Hb (mg/dl)	13 ± 1	13 ± 1
Creatinine (mg/dl)	1.1 ± 0.3	1.1 ± 0.3
Sodium (mEq/l)	138 ± 17	141 ± 2
Potassium (mEq/l)	4.2 ± 0.4	4.1 ± 0.4
Glucose (mg/dl)	95 ± 23	96 ± 25
HbA1c (mmol)	39 ± 8	38 ± 6
Uricemia (mg/dl)	7.2 ± 1.6	7.4 ± 1.6
HDL (mg/dl)	43 ± 11	44 ± 11
Triglyceride (mg/dl)	140 ± 60	130 ± 54
LDL (mg/dl)	112 ± 33	108 ± 30
BNP (pg/ml)*	729 ± 625	274 ± 230

*p < 0.001

*SBP* = systolic blood pressure, *DBP* = diastolic blood pressure, *HR* = heart rate, *LVEF* = left ventricular ejection fraction, *Hb* = haemoglobin, *HbA1c* = glycated haemoglobin, *HDL* = high density lipoprotein, *LDL* = low density lipoprotein, BNP = brain natriuretic peptide

**Table 2:** Cardiologic and respiratory therapy at baseline and after 6 months of treatment (all *p* = not significant)

	Baseline (%)	6 months (%)
Cardiologic therapy		
ACE inhibitors	27	29
ARBs	34	32
Beta-blockers	82	79
Ivabradine	9	9
Digitalis	14	14
Diuretics	95	93
MRAs	57	57
Ca channel blockers	18	16
Statins	36	41
Antiplatelet	63	63
Anticoagulant	45	45
Respiratory therapy		
No therapy	11	0
Titropium	39	0
Glycopyrronium	21	0
Aclidinium	1	0
Indacaterol	4	0
Salmeterol/fluticasone 50/250 μg	1	0
Salmeterol/fluticasone 50/500 μg	3	0
Fluticasone/vilanterol	13	0
Budesonide/formeterol	1	0
Tiotropio/budesonide/formeterol	4	0
Tiotropio/salmeterol/fluticasone	1	0
Aclidinium/fluticasone/vilanterol/theopylline	1	0

*ACE* = angiotensin-converting enzyme, *ARB* = angiotensin receptor blocker, *MRA* = mineralocorticoid receptor antagonist

**Table 3:** Mean values of the most relevant echocardiographic parameters in patients with HfrEF and in those with HFpEF

Echocardiography parameter	Baseline assessment	After 6 months of treatment	*P*-value
Patients with HFrEF (*n* = 16)			
LVEF (%)	34 ± 6	50 ± 14	*p* < 0.001
LVEDV, mL	142 ± 29	122 ± 67	*p* < 0.01
LVESV, mL	81 ± 23	78 ± 20	ns
RVSP, mmHg	36 ± 9	33 ± 8	*P* < .001
Patients with HFpEF (*n* = 40)			
LVEF (%)	58 ± 7	61 ± 7	ns
LVEDV, mL	104 ± 31	92 ± 22	ns
LVESV, mL	41 ± 20	39 ± 8	ns
RVSP, mmHg	36 ± 11	32 ± 9	*P* < .001

*HFrEF* = heart failure with reduced ejection fraction, *LVEF* = Left ventricular ejection fraction, *LVEDV* = left ventricular end-diastolic volume, *LVESV* = left ventricular end-systolic volume, *RVSP* = right ventricular systolic pressure, *HFpEF* = heart failure with preserved ejection fraction, *ns* = not significant

**Table 4:** Mean values of respiratory parameters at baseline and after 6 months of treatment

Parameters	Baseline	6 months	*P*-value
FEV1 (%)	60 ± 6	66 ± 6	*p* < 0.002
TI (%)	60 ± 7	77 ± 4	*p* < 0.001
CAT	19 ± 4	13 ± 3	*p* < 0.001

*FEV1* = Forced expiratory volume at 1 s, *TI* = tiffenau Index

As it happened for echocardiographic parameters (Table 3) improving significantly during treatment, BNP levels were significantly reduced in all patients after 6-month of treatment with IND/GLY compared with baseline levels (*p* = 0.001), and a high percentage of patients improved their clinical status as reflected by the NYHA class status compared with the baseline (*p* < 0.001).

Several minor changes in other parameters assessed were noted, although no statistical significance was evident for blood pressure values, heart rate and hematic parameters. Respiratory parameters showed the following variations: mean FEV1 increased from 60 to 66% (*p* < 0.001), mean TI increased from 60 to 77% (*p* < 0.001) and mean CAT score decreased from 19 to 13 points (*p* < 0.001). In details, the multivariate analysis revealed that patients in NYHA class III showed the best improvements in respiratory parameters: in this subgroup mean TI increased from 59 to 77 (*p* < 0.001), and FEV1 increasing to 69% (*p* < 0.002).

No hospitalization for acute HF or COPD exacerbation occurred during the 6-month period. None of the patients reported adverse events with the new drug, and it was not necessary to discontinue therapy. One patient died during the 6-month follow-up for respiratory failure.

## Discussion

Data from this retrospective analysis, although obtained in a limited case-series, suggest that the combined IND/GLY bronchodilation therapy may be potentially suitable to improve respiratory function in patients with stable COPD and HF. Sample size calculations may have improved the reliability of these results, which are, however, quite significant, no assumptions can be made about them given the retrospective nature of our study. For instance, we noted that the hospitalization rate was strongly reduced during the 6 months of treatment with IND/GLY, and we may speculate that this effect is linked to physiology. In fact, a drug that improves respiratory function may exert relevant benefits also on the cardiovascular system, improving patients’ overall clinical condition. Of note, after 6 months of treatment with IND/GLY, we found that BNP levels were significantly lower compared with baseline values, and the percentage of patients in NYHA class II significantly increased, both signs of an improved clinical status of the patients. Further investigations on the mechanistic base of this potential effect may be interesting to pursue.

A statistically significant increase in EF and a reduction of the end-diastolic volume (EDV) was noted in HFrEF patients after 6-month of treatment with IND/GLY. On the contrary, no statistically significant differences in LVEF was noted for HEpEF; this is not surprising as the mean value of LVEF for these patients was within the normal range of values, so no improvement of EF was expected for the majority of these patients. At the same time, a favourable trend towards improvement of EDV and end-systolic volume (ESV) was noted in this population, so it could also be a matter of insufficient statistical power due to the small sample size. Further studies are required in this population to confirm (or not) these preliminary but very attractive results.

Furthermore, there are data in literature about the use of ICS and LABA/LAMA in patients with myocardial infarction and concomitant COPD. These patients are at increased risk of poor clinical outcomes, including death, as compared to patients without COPD. Contoli et al. showed that in COPD patients, treatment with ICS/LABD may reduce the severity of clinical manifestations during the acute phase of a STEMI, compared to other inhaled treatments [25]. These results may also apply during HF exacerbations. In fact, importantly no severe hemodynamic consequences related to the dual bronchodilation therapy were registered in patients with atrial fibrillation, possibly because they were treated with selective beta 1 receptor blockers and for the reassuring safety of the association of a LABA and a LAMA.

Barr et al. have shown that emphysema and more severe airflow obstruction were linearly related to impaired cardiac function without impacting the EF [26]. Watz et al. have shown that that an increasing severity of COPD, according to the GOLD classification, is associated with a decreasing heart size [27]. Stone, in a short-term study, have shown that pharmacologic treatment of COPD has beneficial effects on cardiac function, but without altering the EF of either ventricle [28]. More related with our investigations are the results of the recently presented CLAIM study [29]; in particular, the study evaluated the effect of IND/GLY on the cardiac function of hyperinflated COPD patients without significant CVD. After 14 days, patients receiving the LABA/LAMA treatment showed a significant increase of the LV and RV EDV, a significant increase of the stroke volume of both ventricles; the reduction of the residual volume correlated with the improvement of EDV, showing a direct effect of the pulmonary therapy on the cardiac parameters. We think our evidence is complementary and supporting these data as we evaluated only patients with concomitant HF. We already know that exacerbation of respiratory symptoms in COPD patients may not be exacerbations of COPD, being potentially caused by exacerbation of coexistent respiratory or non-respiratory diseases (e.g. cardiovascular reasons) [30], so the potential to improve the non-respiratory causes of symptoms worsening with the double bronchodilator therapy looks very attractive. The rationale behind the double bronchodilation in COPD patients with and without HF patients is quite strong, a near maximal bronchodilation and the associated reduction of hyperinflation could be very useful in determining an increase of the venous filling of the heart, thus causing an increase of the compliance and cardiac output. At the same time, some concerns regarding the cardiac safety of the double bronchodilation are easily imaginable, but the data here provided are supportive of the safety of the IND/GLY association, as no HF hospitalization occurred in the entire sample of patients with HF and COPD, which is, if confirmed, a very strong rationale for widely applying this therapeutic strategy, even if a close monitoring of every treatment administered to such a fragile population is of course mandatory. The tolerability of the double bronchodilation appeared very reassuring as no patients stopped the double bronchodilator treatment for side effects. One patient died during the 6-month follow-up, the cause was not related with the any of the administered treatments.

We think our analysis has several strengths, it was conducted on a homogeneous population with HF and COPD, the single centre management of the patients for the instrumental and clinical management, as well as cardiovascular and respiratory assessment reduced to null the variability related with different physician/operator. Echocardiographic measurements were done by the same skilled physician, although we acknowledge that precision of the echocardiographic measurement of cardiac volumes is low when compared to magnetic resonance imaging (MRI), the gold standard for these measurements.

Limitations of the analysis are related with the retrospective nature of the study, which also lacks a proper sample size calculation. A prospective study should be performed to confirm our data in the same population.

## Conclusion

In conclusion, this analysis supports the use of IND/GLY in patients with concomitant HF and COPD, since a statistically significant clinical improvement in respiratory and cardiologic parameters with a reassuring tolerability were noted.

In our opinion, further research in this field is important, to reach a better understanding of the pathophysiologic mechanisms of COPD and HF interrelation, and also to improve on a constant basis the pool of treatments for this complex clinical entity, possibly aiming to eventually reduce morbidity and mortality.

## Abbreviations

COPD: Chronic obstructive pulmonary disease
HF: Heart failure
IND/GLY: Indacaterol/glycopirronium
NYHA: New York Heart Association
SBP: Systolic blood pressure
DBP: Diastolic blood pressure
HR: Heart rate
LVEF: Left ventricular ejection fraction
Hb: Haemoglobin
HbA1c: glycated haemoglobin
HDL: High density lipoprotein
LDL: Low density lipoprotein
BNP: Brain natriuretic peptide
ACE: Angiotensin-converting enzyme
ARB: Angiotensin receptor blocker
MRA: Mineralocorticoid receptor antagonist
HFrEF: Heart failure with reduced ejection fraction
LVEDV: Left ventricular end-diastolic volume
LVESV: Left ventricular end-systolic volume
HFpEF: Heart failure patients who show a preserved ejection fraction
ns: not significant
MRI: Magnetic resonance imaging
CVD: Cardiovascular disease
FEV1: Forced expiratory volume in 1 s
FVC: Forced vital capacity
CAT: COPD Assessment
TI: Tiffenau Index
